# Bacterial Cell–Cell Communication in the Host via RRNPP Peptide-Binding Regulators

**DOI:** 10.3389/fmicb.2016.00706

**Published:** 2016-05-20

**Authors:** David Perez-Pascual, Véronique Monnet, Rozenn Gardan

**Affiliations:** Micalis Institute, INRA, AgroParisTech, Université Paris-Saclay, Jouy-en-JosasFrance

**Keywords:** quorum sensing, firmicutes, virulence, commensalism, cell–cell communication

## Abstract

Human microbiomes are composed of complex and dense bacterial consortia. In these environments, bacteria are able to react quickly to change by coordinating their gene expression at the population level via small signaling molecules. In Gram-positive bacteria, cell–cell communication is mostly mediated by peptides that are released into the extracellular environment. Cell–cell communication based on these peptides is especially widespread in the group Firmicutes, in which they regulate a wide array of biological processes, including functions related to host–microbe interactions. Among the different agents of communication, the RRNPP family of cytoplasmic transcriptional regulators, together with their cognate re-internalized signaling peptides, represents a group of emerging importance. RRNPP members that have been studied so far are found mainly in species of bacilli, streptococci, and enterococci. These bacteria are characterized as both human commensal and pathogenic, and share different niches in the human body with other microorganisms. The goal of this mini-review is to present the current state of research on the biological relevance of RRNPP mechanisms in the context of the host, highlighting their specific roles in commensalism or virulence.

## Introduction

Throughout the human body, all surfaces with portals to the exterior are covered in microbes (Foxman and Martin, 2015), mostly bacteria, which adapt to the specific environmental conditions of each niche by fighting, competing, or co-habiting with other bacteria and host cells.

In these kinds of complex consortia, bacteria have developed sophisticated communication mechanisms wherein signaling molecules are exported into the extracellular environment, accumulated, and then detected by a sensor protein. This sensing leads bacteria to modulate the expression of target genes, thus enabling them to escape from immune defense or attack the host (in the case of virulence) or compete with other bacteria (in commensalism).

In Firmicutes, cell–cell communication, or quorum sensing, is mostly mediated by peptides. Most of these peptides are encoded by short open reading frames but a few of them are produced by the proteolytic degradation of signal peptides of lipoproteins. All of them are then released into the extracellular environment (Antunes and Ferreira, 2009; Atkinson and Williams, 2009). Some of these peptides can be detected in the extracellular medium by two-component systems, while others are actively imported back into bacteria by the oligopeptide transporter Opp, at which point they interact with sensors in the cytoplasm. This interaction modulates the activity of the sensors and thus the expression of target genes (Rocha-Estrada et al., 2010; Cook and Federle, 2014; Monnet and Gardan, 2015). These sensors belong to the RRNPP family of cytoplasmic regulatory receptors (name given from the different sensors described: Rgg, Rap, NprR, PlcR, and PrgX). They are characterized at the structural level by tetratricopeptide repeats, which are involved in the regulator/peptide interaction (Declerck et al., 2007; Parashar et al., 2015). Rgg, NprR, PlcR, and PrgX are transcriptional regulators *sensu stricto* and will be examined in this review (**Table 1**), whereas the Rap phosphatases found in bacilli will not. RAP proteins have a phosphatase activity and regardless of this catalytic activity, they can also inhibit the transcriptional activity of response regulators of two component systems. They have been mainly studied in *Bacillus subtilis* and are involved in the control of sporulation, competence, and production of degradative enzymes and antibiotics (for review see Pottathil and Lazazzera, 2003).

**Table 1:** RRNPP family of transcriptional regulators with their associated peptides and their biological roles.

Name of regulator	Peptide	Group	Role of mechanism
Rgg	SHP, ComS (XIP)	Streptococci	Commensalism/Virulence
NprR	NprX	Bacilli	Necrotrophism
PlcR	PapR	Bacilli	Virulence
PrgX, TraA	cCF10 iCF10	Enterococci	Virulence
TprA	PhrA	Streptococci	Commensalism

**FIGURE 1:**
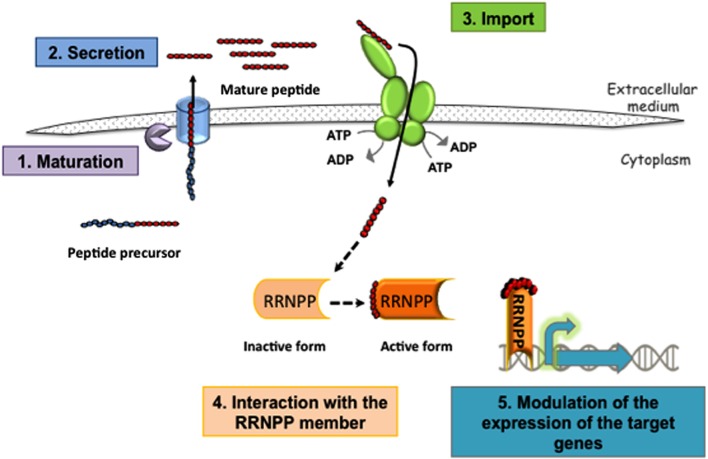
**Schematic representation of the RRNPP mechanism involving transcriptional regulators.** (1) Maturation of a peptide precursor (or protein in *E. faecalis*) and (2) secretion of the mature peptide releases a signaling peptide outside the bacterium. These two processes involve various exporters and proteases, depending on the bacteria, and some have yet to be identified. The order in which these steps occur is also largely unknown. (3) Next, the signaling peptide is imported into the cell by Opp, a transporter from the ATP-binding cassette (ABC) family. (4) Finally, the signaling peptides interact with their specific sensors (Rgg, NprR, PlcR, or PrgX), and (5) modulate the expression of their target genes. For NprR, PlcR, or the Rgg which function as activators binding to their cognate peptide allows them to positively control the expression of their target genes. For TprA or the Rgg3 that function as repressor binding to their cognate peptide alleviates the repressor effect. The repressing function of PrgX is either strengthened by the binding of an inhibitory peptide or weakened by the binding of an activating peptide.

Different RRNPP mechanisms have been deciphered in detail (**Figure 1**) and described in several recent reviews (Cook et al., 2013; Slamti et al., 2014; Fontaine et al., 2015). However, these mechanisms have been validated mostly *in vitro*, and very few studies have explored how they function in complex environments. The RRNPP members that have been studied so far, are found in bacilli, streptococci, or enterococci. Some of the species of interest are human opportunistic pathogens and share different niches in the human body with other bacteria. This mini-review will focus on the data available concerning the functioning of these mechanisms in the context of the host. In addition, we will examine whether these systems are linked to virulence or commensalism.

## PrgX/TraA Repressors and Virulence in Enterococci

Conjugation is a horizontal gene transfer mechanism that controls the transfer of genes from donor cells to recipient cells after contact. Conjugation of sex pheromone plasmids in *Enterococcus faecalis* is controlled by a transcriptional repressor in the PrgX/TraA family that is able to bind either an activating or a repressing peptide. The conjugation of the pCF10 plasmid has been studied in detail. Very briefly, when bound to the activating peptide cCF10, PrgX adopts a dimeric form, which allows the expression of *prgQ* and the conjugation genes. In this way, conjugation can occur between pCF10 of the donor and the recipient cell. Instead, when bound to the inhibitory peptide iCF10, the PrgX repressor adopts a tetrameric conformation; this represses the transcription of *prgQ*, which encodes iCF10, as well as that of downstream conjugation genes. Therefore, the conjugation cannot occur (**Figure 1**) (Clewell et al., 2014). This mechanism and additional regulatory pathways at the transcriptional and post-transcriptional levels have been thoroughly described in multiple recent reviews (Dunny, 2007, 2013; Dunny and Johnson, 2011).

*E. faecalis* is a commensal bacterium that colonizes the intestinal tract of humans as well as the vagina and oral cavity, but it is also an opportunistic pathogen which causes significant nosocomial infections. Over 20 plasmids belonging to the sex pheromone family have been found to be more prevalent in clinical enterococcal strains (Coque and Murray, 1995). *In vivo* conjugation has been reported in human plasma (Hirt et al., 2002) as well as in different animal models such as the gastrointestinal tract of hamsters, rabbit plasma, and the endocarditis or subdermal chamber in rabbits (Huycke et al., 1992; Hirt et al., 2002).

Part of the regulon of the PrgX system is the aggregation substance (AS), a surface protein that mediates donor/recipient attachment and which is encoded by the *prgB* gene. Interestingly, synthesis of AS is induced, through the PrgX mechanism, in donor cells in contact with human plasma, in the absence of recipient cells (Hirt et al., 2002). This observation led to the proposal of a model in which a plasma component, probably an albumin/lipid complex, inactivates the inhibitory peptide iCF10, thus allowing the expression of the conjugation genes, including *prgB* (Chandler et al., 2005). Beyond its role in conjugation, AS has been shown to be a virulence factor in different rabbit models of endocarditis; in some of these models it produces cardiac vegetations and higher mortality (Chow et al., 1993; Schlievert et al., 1998; Hirt et al., 2002). AS also accelerates early biofilm development in an *ex vivo* porcine heart valve model (Chuang-Smith et al., 2010). It promotes adhesion to cultured renal tubular cells from pork (Kreft et al., 1992) and adhesion to and invasion of different types of cultured human enterocytes (Olmsted et al., 1994; Sartingen et al., 2000; Wells et al., 2000; Waters et al., 2003, 2004). Finally, AS also protects *E. faecalis* against attack by human neutrophils despite phagocytosis and neutrophil activation (Rakita et al., 2000) and by human macrophages (Sussmuth et al., 2000).

## PlcR/PapR and Virulence in the *Bacillus cereus* Group

The *B. cereus* group comprises multiple species (Bottone, 2010), including the insect pathogen *Bacillus thuringiensis*, the etiological agent of anthrax *Bacillus anthracis*, (Kolsto et al., 2009), and *B. cereus sensu stricto*, an opportunistic pathogen causing food-borne gastroenteritis and systemic infections (Stenfors Arnesen et al., 2008). In *B. cereus* and *B. thuringiensis*, PlcR is a pleiotropic transcriptional activator that induces the expression of 80% of the genes coding for extracellular factors, including some virulence factors such as degradation enzymes and cytotoxic and cell surface proteins (Agaisse et al., 1999; Gohar et al., 2002, 2008). Interaction of the peptide PapR with PlcR allows the complex to bind to DNA and enables transcription of the target genes (**Figure 1**) (Grenha et al., 2013). In *B. thuringiensis*, disruption of the *papR* gene inactivated the expression of the PlcR regulon, resulting in decreased hemolytic activity and a significant reduction in virulence in an insect infection model (Slamti and Lereclus, 2002).

Other studies have also identified a role for PlcR in the regulation of pathogenesis in *B. cereus* and *B. thuringiensis*. In both of these bacteria, PlcR inactivation provokes a significant reduction in virulence in mice (Salamitou et al., 2000) and rabbits (Callegan et al., 2003). Similarly, in *ex vivo* cell culture assays, cytotoxicity of *B. thuringiensis* strain Bt407 in Hela or Caco-2 human epithelial cells was PlcR-dependent. In addition, this toxic effect was cell-contact-independent, which supports the hypothesis that at least one of the secreted factors regulated by PlcR is responsible for this cytotoxicity (Ramarao and Lereclus, 2006). Three important enterotoxins in the *B. cereus* group – the hemolysin BL (Hbl), the cytotoxin K (CytK), and the non-hemolytic enterotoxin (Nhe) – are under control of the PlcR–PapR system (Agaisse et al., 1999). Hbl and Nhe are pore-forming toxins, which induce cell lysis in different eukaryotic cell models (Jessberger et al., 2014) and CytK is cytotoxic toward the Caco-2 intestinal cell line (Hardy et al., 2001; Fagerlund et al., 2004; Jessberger et al., 2014). More generally, it is hypothesized that these enterotoxins are responsible for the abdominal cramps and diarrhea that are symptoms of infection (Ramarao and Sanchis, 2013). However, *plcR* mutant strains are not completely avirulent (Tran et al., 2011), suggesting that other virulence factors non-regulated by PlcR play a role during *B. cereus* infections (Guillemet et al., 2010).

## NprR/NprX and Necrotrophism in the *B. cereus* Group

The NprR/NprX system appears to be functional in three of the species in this group: *B. anthracis*, *B. cereus*, and *B. thuringiensis* (Rice et al., 2015). The sensor NprR interacts with the signaling peptide NprX, and the complex then activates the expression of NprA metalloprotease (Perchat et al., 2011), as well as other genes that encode degradative enzymes like lipases or peptidases (**Figure 1**) (Dubois et al., 2012). Interestingly, the PlcR–PapR complex stimulates the transcription of the *nprR–nprX* operon (Dubois et al., 2013).

In *B. thuringiensis*, it has been proposed that this system participates in the necrotrophism process in an insect larval model (Dubois et al., 2012). However, little is known about its activity in the mammalian environment, with one exception: in *B. anthracis*, the *nprR* gene was highly expressed during the outgrowth of spores within murine macrophages (Bergman et al., 2007), which suggests that this system plays a role in *B. anthracis* pathogenesis.

## Rgg/SHP in Commensalism and Virulence in Streptococci

The Rgg transcriptional regulators are widespread among many species of the phylum Firmicutes. However, Rgg-associated peptides have only been found in Streptococcaceae thus far. Two subfamilies of Rgg are distinguished: (i) Rgg associated with the SHP (small hydrophobic peptides) family of peptides, which regulates genes of diverse functions, and (ii) the Rgg called ComR, which, in association with the ComS (XIP) family of peptides, controls the triggering of competence in multiple species (Ibrahim et al., 2007b; Fleuchot et al., 2011).

Peptide-associated Rgg mechanisms were first deciphered in *Streptococcus thermophilus* (Ibrahim et al., 2007a; Fontaine et al., 2010, 2013; Fleuchot et al., 2011) and later in three opportunistic pathogenic streptococci (**Figure 1**). The first of these was the human commensal associated with dental caries, *Streptococcus mutans*, which contains a ComS/ComR system (Mashburn-Warren et al., 2010). To our knowledge, no data are available *in vivo* for this bacterium. The second was the human-restricted pathogen *Streptococcus pyogenes* (or GAS, from Group A *Streptococcus*), which has two interrelated SHP/Rgg systems, SHP2/Rgg2 and SHP3/Rgg3, in addition to a ComS/ComR system (Chang et al., 2011). More recently, the opportunistic human and animal pathogen *Streptococcus agalactiae* (or GBS, from Group B *Streptococcus*; Cook et al., 2013; Perez-Pascual et al., 2015) was determined to have one active SHP/Rgg, called SHP/RovS.

GAS is commonly carried asymptomatically in the oropharynx or on the skin but can become invasive and cause severe life-threatening diseases. The *shp2/rgg2* and *shp3/rgg3* loci are present in all sequenced strains of GAS and the complex Rgg2/3 pathway of strain NZ131 has recently been extensively studied in chemically defined medium (Lasarre et al., 2013; Cook and Federle, 2014). In addition to the *shp2/3* genes, Rgg2/3 control the transcription of downstream genes whose function remains unclear. However, this pathway modulates the levels of biofilm produced by GAS (Chang et al., 2011). Moreover, SHP/Rgg signaling is induced considerably in the presence of mannose, one of the prominent carbohydrates present in the glycoconjugates on airway epithelia. In addition, these inducing conditions confer upon GAS resistance to lysozyme, an antimicrobial host defense mechanism present in mucosal secretions and in macrophages (Chang et al., 2015). Additional experiments also showed that the peptidase PepO, whose gene is negatively regulated by CovR, can degrade SHP2 and SHP3 and, therefore, inhibit SHP/Rgg signaling. CovR is a two-component system regulator that inhibits the expression of many genes, including those encoding potential virulence factors. Despite the lack of direct *in vivo* experimental evidence for the biological relevance of these SHP/Rgg systems in GAS, these results led the authors to hypothesize that the Rgg2/3 pathway more likely plays a role in asymptomatic carriage in the host (Wilkening et al., 2015). However, in contrast to this hypothesis, another study in strain SF370 demonstrated that Rgg2 and its associated regulon were implicated in infection development, as the inactivation of *rgg2* decreased the ability of GAS to adhere to epithelial cells and increased biofilm formation. Additionally, a Δ*rgg2* mutant strain was unable to grow in human blood and showed avirulent behavior in a murine model (Zutkis et al., 2014).

GBS is a commensal of the human intestinal and vaginal tract in 15–30% of healthy adults, but remains one of the most important invasive pathogens in newborns and the elderly (Le Doare and Heath, 2013). Nearly all sequenced GBS strains present a unique copy of the *shp*/*rovS* locus. This cell–cell communication mechanism has been recently deciphered (Cook et al., 2013; Perez-Pascual et al., 2015), demonstrating that the SHP/RovS mechanism plays an important role in bacterial pathogenesis. Mice infected by isogenic *shp* or *rovS* deletion mutants showed a significant decrease in the bacterial burden in the liver and spleen compared to mice infected by the parental strain. Further exploration revealed that disruption of *shp* and *rovS* resulted in a significant decrease in the ability of GBS to adhere to and invade human HepG2 hepatic cells (Perez-Pascual et al., 2015). In addition, in chemically defined medium, at least three genes are under the control of this mechanism: *shp* and *gbs1556* (encoding a secreted protein), which are positively and directly controlled by SHP/RovS, and the *fbsA* gene, which codes for a fibrinogen-binding protein involved in GBS pathogenesis (Schubert et al., 2004) and is repressed by SHP/RovS. In rich medium, RovS also seems to control a set of genes related to virulence, such as *sodA* and *cylE*, which encode a superoxide dismutase and a protein required for the production of hemolysin, respectively (Samen et al., 2006).

Another peptide-associated Rgg transcriptional regulator has been studied in *Streptococcus suis* (Zheng et al., 2011). In this bacterium, inactivation of Rgg attenuated pathogenicity in a piglet infection model, probably due to growth and metabolism changes in the mutant. Surprisingly, a Δ*rgg* mutant showed hyper-adhesion to epithelial cells and increased hemolytic activity. DNA microarray analysis revealed that Rgg is a global regulator that affects genes with varied functions, such as the metabolism of non-glucose carbohydrates (e.g., lactose or maltose) or defense mechanisms. The authors suggest that Rgg is a global transcriptional regulator that plays a relevant role in *S. suis* survival during pathogen–host interaction (Zheng et al., 2011).

In streptococci, the *comX* gene encodes an alternative sigma factor that controls the transcription of the genes necessary for natural transformation, and transcription of *comX* is either controlled by a two-component system or a peptide-associated Rgg-like regulator, the ComS/ComR system. The involvement of ComS/ComR in the triggering of transformation has been experimentally validated in laboratory conditions for four different species: *S. mutans*, *S. thermophilus*, *Streptococcus infantarius*, and *Streptococcus macedonicus* (Fontaine et al., 2015). Interestingly, in GAS, in spite of the fact that no natural transformation has been observed in many laboratory conditions (Mashburn-Warren et al., 2012), transformants were recovered at low levels from cells grown in biofilm on epithelial cells or *in vivo* from mice colonized intranasally with biofilm bacteria (Marks et al., 2014).

## TprA/PhrA and Commensalism in *Streptococcus pneumoniae*

Recently, new families of peptide-associated regulators have been identified in *S. pneumoniae*, which is commensal in the human nasopharynx, but also the etiological agent of serious diseases such as pneumonia, bacteremia, or meningitis (Shak et al., 2013). These new families comprise a transcriptional regulator, Tpr, and its allied cognate peptide Phr (Hoover et al., 2015). Blast analysis revealed that this system appears to be orthologous to the PlcR/PapR system in bacilli. One such system, TprA/PhrA, regulates the expression of a putative lantibiotic biosynthesis operon. TprA represses the expression of its own encoding gene, as well as that of the *phrA* gene and the lantibiotic genes. However, the PhrA peptide antagonizes TprA activity. A study of this regulation following the expression of the *phrA* gene revealed that, whereas glucose can inhibit *phrA* expression, galactose, the main sugar of the human nasopharynx, can induce it (Hoover et al., 2015). Tn-seq analysis (sequencing of insertion transposon sites by high throughput sequencing methods) highlighted that disruptions of *tprA* or some of the lantibiotic genes reduced the fitness of *S. pneumoniae* for nasopharynx colonization in a murine model (van Opijnen and Camilli, 2012). These results led Hoover et al. (2015) to hypothesize that expression of the lantibiotic genes through the TprA/PhrA system may be important during niche colonization, where *S. pneumoniae* competes with other bacteria for space and resources. This was supported by the observation that the TprA/PhrA system and its regulated lantibiotic gene cluster are not needed for invasive disease development in a murine model (van Opijnen and Camilli, 2012; Hoover et al., 2015).

## Conclusions and Perspectives

In Firmicutes, peptides are the best-known signaling molecules. The peptides that interact with RRNPP regulators are short (< 10 amino acids), without post-translational modifications, are secreted and then reimported into bacteria through an oligopeptide transporter. The RRNPP transcriptional regulators are activated by these peptides via tetratricopeptide domains involved in peptide-protein interactions. RRNPP regulators and reimported short peptides act together as a communication system, which clearly differs from a second group of communication systems. In this second group, which includes the well-documented Agr system from *Staphylococcus aureus*, peptides are post-translationally modified and activate two components systems at the outside bacterial surface.

The number of putative members of the RRNPP family has increased dramatically; for example, in streptococci, the Tpr/Phr and Rgg/SHP families are predicted to have 53 and 68 members, respectively, (Fleuchot et al., 2011; Hoover et al., 2015) and it is highly probable that others remain to be discovered. In order to better understand these regulators, much work remains, especially focusing on their role and *in vivo* function in opportunistic pathogens. Research conducted thus far indicates that RRNPP members regulate a variety of biological functions. For example, PlcR/PapR as well as PrgX/TraA have a direct impact on virulence, through controlling the expression of target genes that encode virulence factors. The TprA/PhrA system has been implicated in the commensal behavior of *S. pneumoniae* and seems to be involved in the expression of a bacteriocin-encoding operon. The role of Rgg/SHP mechanisms is more complex. Whereas the RovS/SHP system in GBS seems to participate in disease development, the orthologous mechanism in GAS, Rgg2/SHP2, has been predominantly linked to commensal behavior. Moreover, this system is expressed at variable levels in different strains of GAS (Chang et al., 2015).

Existing conclusions regarding the regulatory function of RRNPP members in the host are derived from indirect results and require further validation. In particular, this field would benefit from *in vivo* imaging of the induction of these mechanisms during host colonization. Furthermore, gene expression studies at the single-cell level are rare; to our knowledge, only one such study exists, an investigation of PlcR and NprR of *B. thuringiensis* in an insect model (Verplaetse et al., 2015). These innovative approaches should greatly improve the identification of host-associated environmental conditions that induce these mechanisms.

Finally, when viewed in the context of emerging resistance to antibiotics, the RRNPP systems represent an attractive target for research into new mechanisms for bacterial control. In the future, it is possible that infections by some opportunistic pathogens could be prevented by decreasing the expression of virulence factors, without altering the composition of the global microbiota, via modulation of the activity of RRNPP regulators.

## Author Contributions

All authors listed, have made substantial, direct and intellectual contribution to the work, and approved it for publication.

## Conflict of Interest Statement

The authors declare that the research was conducted in the absence of any commercial or financial relationships that could be construed as a potential conflict of interest.
